# Targeting JP2: A New Treatment for Pulmonary Hypertension

**DOI:** 10.1155/2021/2003446

**Published:** 2021-08-06

**Authors:** Rubin Tan, Cui Li, Chuan Xu, Qi Wu, Liping Gao, Yue Shi, Jie Cui

**Affiliations:** ^1^Department of Physiology, Xuzhou Medical University, Xuzhou 221004, China; ^2^Department of Nosocomial Infection Management, Tongji Hospital, Tongji Medical College, Huazhong University of Science and Technology, Wuhan 430030, China

## Abstract

Pulmonary hypertension (PH) is a disease with a complex etiology and high mortality rate. Abnormal pulmonary vasoconstriction and pulmonary vascular remodeling lead to an increase in mean pulmonary arterial blood pressure for which, and there is currently no cure. Junctophilin-2 (JP2) is beneficial for the assembly of junctional membrane complexes, the structural basis for excitation-contraction coupling that tethers the plasma membrane to the sarcoplasmic reticulum/endoplasmic reticulum and is involved in maintaining intracellular calcium concentration homeostasis and normal muscle contraction function. Recent studies have shown that JP2 maintains normal contraction and relaxation of vascular smooth muscle. In some experimental studies of drug treatments for PH, JP2 expression was increased, which improved pulmonary vascular remodeling and right ventricular function. Based on JP2 research to date, this paper summarizes the current understanding of JP2 protein structure, function, and related heart diseases and mechanisms and analyzes the feasibility and possible therapeutic strategies for targeting JP2 in PH.

## 1. Introduction

Pulmonary hypertension (PH) is characterized by increased mean pulmonary arterial blood pressure (mPAP), and right ventricular hypertrophy (RVH) [1, 2], and causes significant morbidity and mortality despite much recent therapeutic progress. The sixth World Health Organization updated the clinical classification of PH based on common pathological features, hemodynamics, and treatment methods. PH was divided into five groups according to the occurrence of several different disease states, including pulmonary arterial hypertension (PAH, group 1), PH due to left heart diseases (PH-LHD, group 2), PH due to lung diseases and/or hypoxia (PH-LD/H, group 3), chronic thromboembolic PH (CTEPH, group 4), and PH with unclear and/or multifactorial mechanisms (group 5) [1]. Elevations of mPAP are caused by persistent pulmonary vasoconstriction and pulmonary vascular remodeling in patients with PH and are mainly triggered by an increase of [Ca^2+^]_i_ in pulmonary arterial endothelial cells (PAECs) and pulmonary arterial smooth muscle cells (PASMCs) [3, 4]. Increased [Ca^2+^]_i_ levels have been confirmed in PAECs under hypoxia [5] and in PASMCs from patients with idiopathic PH (IPAH) [3, 6–9].

All types of muscle contraction are performed through excitation-contraction (E-C) coupling, that is, when the excitation is transmitted to the plasma membrane (PM), the PM depolarizes, resulting in the opening of voltage-gated calcium channels (VDCCs) and the entry of extracellular calcium, activating ryanodine receptors (RyRs) on the sarcoplasmic reticulum/endoplasmic reticulum (SR/ER), and resulting in calcium release by the SR/ER. This is also known as calcium-induced calcium release (CICD). An increase in [Ca^2+^]_i_ leads to muscle contraction. For many years, researchers have focused on the ion channels involved in action potentials, especially the calcium channels involved in muscle contraction. It was later found that junctional membrane complexes (JMCs) composed of VDCC on PM and RyRs on SR/ER are the structural basis for E-C coupling [10]. Takeshima et al. reported that junctophilins (JPs) located between VDCC on the PM and RyRs on the SR/ER contribute to JMC assembly [11]. They are key factors involved in JMC maturation and stability [12], as well as [Ca^2+^]_i_ homeostasis [13–16]. To date, mammals have four JP subtypes (1-4), and different subtypes and/or expression levels differ among the different tissues [17–20].

JP2, the main expression subtype, is abundant in the myocardial and smooth muscles. Every part of the JP2 protein plays a different role in E-C coupling, such as promoting and stabilizing JMCs [12], maintaining intracellular Ca^2+^ homeostasis [13], and being a transcription factor under stress [26]. In addition, many diseases are related to its expression or mutation, and many studies have focused on the treatment of targeted JP2 proteins. This review summarizes the structure and function of JP2 and its related diseases and explores its feasibility as a novel therapeutic target for PH based on the current research results of JP2 in the smooth muscle.

## 2. JP2 Structure

The *JP2* gene is located on chromosome 20 and consists of 2902 bases. The DNA strand is 75.88 kb long, the mRNA strand is 4802 bp, and there are six exons. The JP2 protein contains 696 amino acids, with 92% similarity between humans and rats [18]. The JP2 protein is composed of “membrane occupation and recognition nexus” (MORN) domains in the N-terminal interacting with the cell membrane, an *α*-helix domain spanning space, a divergent domain, and a transmembrane domain (TMD) in the C-terminal anchoring JP2 in SR/ER membrane [21–24].

The MORN motif region (amino acids 14-336) with eight MORN motifs comprises a MORN motif region I (MORN I-VI), a joining region (approximately 120 amino acids, CRD) [25], and a motif region II (MORN VII-VIII) [23, 24]. Each MORN motif region consists of 14 amino acids, with a roughly identical sequence of YQ/EGE/QT-X-NGK-X-HGYG [12, 26]. Three flanking Cys residues in the MORN motifs which are s-palmitoylated are necessary for binding lipid raft domains in PM [27, 28]. The MORN motif mediates binding to the PM by acting on phosphatidylinositol (PI (4,5) P2), which is concentrated in PM. This interaction occurs throughout the sarcolemma, not just in the T-tubule [29]. Amino acids 302–327 may be a PM-binding motif that contains PIP-5 kinase and protein kinase sites [22, 30, 31]. The *α*-helix structure (amino acids 337–430) includes a bipartite nuclear localization signal-like peptide (bNLS, amino acids 345–359), a helical region, an alanine-rich region (ARR, amino acids 367–402), and a putative *α*-helical region, which shows the characteristics of the helix structure [26].

**Figure 1:**
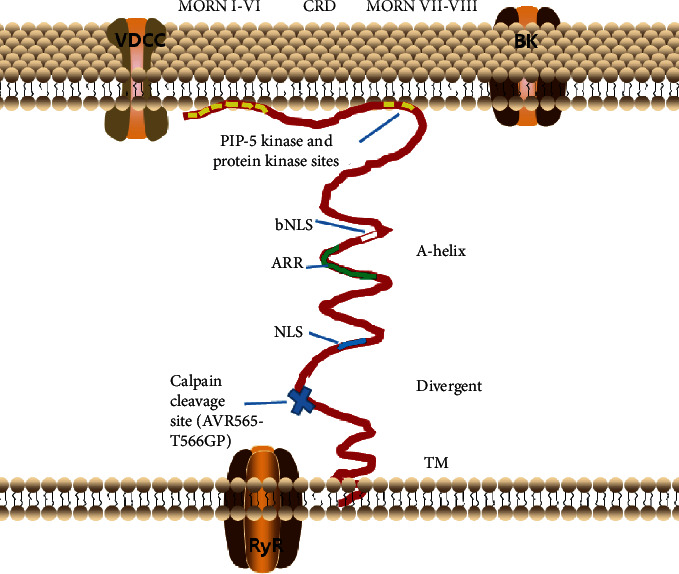
JP2 structure and location in the striated muscle cells. JP2 is located between VDCC and RyRs. JP2 is composed of MORN domains in the N-terminal interacting with the cell membrane, an *α*-helix domain spanning space, a divergent domain, and a transmembrane domain (TMD) in the C-terminal anchoring JP2 in the SR/ER membrane. PIP-5 kinase and protein kinase sites are in the MORN domains, and calpain cleavage site (AVR565-T566GP) is in the divergent domain. ARR: alanine-rich region; BK: big conductance Ca^2+^-dependent K+ channel; bNLS: bipartite nuclear localization signal-like peptide; CRD: joining region; JP2: junctophilin-2; MORN: membrane occupation and recognition nexus; NLS: monopartite nuclear localization signal; RyR: ryanodine receptor; TM: transmembrane domain; VDCC: voltage-dependent Ca^2+^ channel.

The divergent domain has a monopartite nuclear localization signal (NLS, amino acids 488–492) and a primary calpain cleavage site (amino acids 565–566) [26]. TMD (amino acids 674–696) has one palmitoylation of Cys which stabilizes JP2's anchor in the ER/SR membrane, as shown in Figure 1 [27, 28]. In particular, JP1 interacts with JP2 to form homo- and heterodimers at the TMD and cytosolic regions and promotes the JP1 localization [29]. The subcellular localization of JP2 is mediated by microtubules [32, 33].

## 3. JP2 Function in the Heart

### 3.1. Maintaining JMC Stability in the Heart

JMC in the myocardium is mainly composed of VDCC on the T-tubule membrane and RyRs on the SR membrane. Therefore, T-tubule maturity and stability determine JMC function and stability. Studies have indicated that JP2 plays a crucial role in T-tubule maturity during fetal heart development [34–37]. JP2 is also required for T-tubule maturity during the early neonatal phase. JP2 knockdown mice suffer from congestive heart failure [38, 39]. JP2 plays a critical role in T-tubule maturation during the late phase after birth through direct interaction with caveolin-3 (Cav 3) [37, 40, 41]. Although a key protein, it cannot completely block T-tubule maturation when it is lacking. Bridging integrator 1 (Bin1) and Cav 3 may affect T-tubule formation through an independent mechanism [40]. Thus, JP2 has a secondary effect on T-tubule maturation in normal hearts after birth, but it is necessary to stabilize T-tubules in the failing heart [42]. When the *JP2* gene is knocked down in adult hearts, the T-tubule will suffer serious damage, probably because the combination of JP2 and T-tubules can maintain T-tubule stability and prevent its degradation [38, 39]. The T-tubule is a dynamic and adjustable structure that may recover after its destruction. JP2 protein hydrolysis initiation and termination are the molecular mechanisms leading to this important remodeling cycle [43].

### 3.2. [Ca^2+^]i Homeostasis

**Figure 2:**
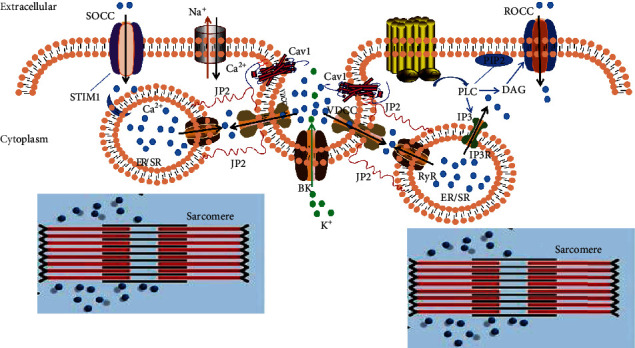
Channel communication in JP2-mediated JMC of smooth muscle cells. There are three main channels for external Ca^2+^ influx in smooth muscle cells: voltage-dependent Ca^2+^, receptor-operated Ca^2+^ entry, and storage-operated Ca^2+^ entry. Internal calcium release occurs mainly from the sarcoplasmic reticulum and endoplasmic reticulum to the cytoplasm through IP3Rs and RyRs. JP2 interacts with Cav 1 and is located in BK channels near RyRs to mediate the crosstalk between them, maintain normal potassium outflow, and cause membrane hyperpolarization, leading to smooth muscle relaxation.

[Ca^2+^]_i_ homeostasis indicates that the increase in [Ca^2+^]_i_ concentration caused by external Ca^2+^ influx from the PM and internal Ca^2+^ release from the SR/ER is consistent with the Ca^2+^ pump uptake of the SR/ER and the Ca^2+^ discharged by the Na^+^-Ca^2+^ exchanger on the PM. In this way, after the cells use the calcium, calcium ions return to the SR/ER and extracellular fluid to keep the [Ca^2+^]_i_ concentration relatively stable. There are three main channels for external Ca^2+^ influx: VDCC, receptor-operated Ca^2+^ entry channel (ROCC), and storage-operated Ca^2+^ entry channel (SOCC), as shown in Figure 2. Internal calcium release occurs mainly from the SR/ER to the cytoplasm through inositol triphosphate receptors (IP3Rs) and RyRs. JP2 can act directly or indirectly on these channels or on the proteins that regulate the activities of these channels to maintain calcium homeostasis. For instance, CICD between VDCC and RyRs occurs in JMCs which are established and maintained by JP2 in the cardiac muscle [14–16, 39]. JP2 can directly act on VDCC to positively modulate the VDCC current [44, 45]. JP2 also directly or indirectly acts on RyR_2_, SR/ER Ca^2+^-ATPase 2a (SERCA2a), triadin, and calsequestrin 2 (CSQ2) to affect Ca^2+^ release and recruit RyR_2_ to the dyads. Triadin and CSQ2 are RyR2 modulators [48, 49]. Furthermore, in the skeletal and cardiac muscle, JP2 can also interact with transient receptor potential canonical (TRPC3) which encodes the repertoire of nonselective cation channels [87]. Recent studies have shown that JPs can also redistribute to the discrete SR/ER region through stromal interaction molecule 1 (STIM1) protein, promoting SOCC. It is found that JP1 and JP2 seem to enhance the activity of SOCC in skeletal muscle synergistically [80, 81]. JP2 can directly act on potassium voltage-gated channel subfamily Q member 1 (KCNQ1) to negatively modulate slow delayed rectifier current [46, 47]. Recently, Fan et al. found that MORN motifs within JP2 can directly act on the small-conductance Ca^2+^-activated K^+^ channels (SK2 channel) in the heart [50]. Some studies showed that JP2 can interact with caveolin-1.1 (Cav 1.1) and Cav 3 involved in [Ca^2+^]_i_ homeostasis [41, 51–53].

### 3.3. Transcription Factor

There is a primary calpain-1 cleavage site in the divergent domain of JP2 between residues R565/T566 [26], so that full-length JP2 can be cleaved into two parts by calpain-1 under stress: one is JP2NT (amino acids 1–565) containing bNLS, ARR, and NLS; the other is JP2CT (amino acids 566-696) containing TMD. Guo et al. reported that NLS is necessary to import JP2NT into the nucleus, and ARR binds to DNA via the TATA box. According to the DNA binding profile, JP2NT is mainly located at the transcription initiation site; therefore, it is considered a transcription regulator [26].

Through the original analysis of differentially expressed genes between JP2NT-overexpressing (JP2NT-OE) mice and wild-type (WT) mice, it was confirmed that JP2NT-OE inhibited some signaling pathways, such as extracellular-regulated mitogen-activated protein kinase (ERK/MAPK), nuclear factor kappa B (NF-*κ*B), transforming growth factor beta (TGF-*β*), and integrin, which are associated with myocardial hypertrophy, fibrosis, cell growth, differentiation, and inflammation. VDCC density, amplitude, and dynamics of Ca^2+^ transients, Ca^2+^ concentrations in SR, and E-C coupling function at the single cell were not changed by JP2NT-OE. After transverse aortic constriction (TAC), JP2NT-OE mice showed improved cardiac function compared with WT mice and attenuated heart failure development by interaction with the myocyte enhancer factor 2 (MEF2) response element (MER) competing for the transcription factor MEF2 family, consisting of master regulators of hypertrophic genes [26].

## 4. JP2-Related Diseases and JP2 Dysregulation Mechanisms

JP2 levels are severely downregulated in patients with hypertrophic cardiomyopathy [54], heart failure [38], and ischemia/reperfusion (I/R) injury [55], elevated wall stress [56], transverse aortic constriction [47, 57], myocardial infarction [58], atrial fibrillation [59], and premature ventricular contraction-induced cardiomyopathy [49]. Decreased JP2 expression in the myocardium has been confirmed in various heart failure models, for instance, in a murine hypertrophic or dilated cardiomyopathy model [41], an I/R injury model [60], a rat hypertrophic cardiomyopathy model [61, 62], a murine myocardial infarction model [63], and a rat PH model [64, 65]. This loss of JP2 expression mainly includes decreased expression and increased proteolysis. Early studies showed that miR-24 combined with JP2 mRNA to downregulated JP2 protein levels [38, 66, 67]. However, a recent study found that an increase in miR-34a led to a decrease in JP2 expression, and even damaged JMC, which further manifested as cardiac insufficiency leading to progressive heart failure [68]. JP2 is cleaved by calpain under stress [69–71]. In 2010, Ali et al. and Ziman et al. found that matrix metallopeptidase 2 (MMP-2) and JP2 colocate the Z-disc of cardiomyocytes [72, 73]. Chan et al. further confirmed that MMP-2 degrades JP2 in the early stage of myocardial I/R injury and mainly acts on amino acids between MORN and divergent regions by using CleavPredict and in vitro proteolysis assays [60]. In addition, the subcellular localization of JP2 was altered in the hypertrophy induced by pressure overload [33] and Duchenne muscular dystrophy [32]. Although JP2 is downregulated in these diseases, Guo et al. found that the cardiac function of JP2-overexpressing mice is not changed under normal conditions but was better than that of WT mice in the development of heart failure after cardiac stress on account of JP2 stabilizing the T-tubule network integrity [88]. Guo et al. also found that JP2NT overexpression attenuates cardiac hypertrophy and heart function after pressure overload and does not affect its normal function mainly because JP2NT can translocate into the nucleus and bind to MER to affect MEF2 transcription under stress, thereby retarding cardiac hypertrophy [26]. Prins et al. also found that right heart function is improved by colchicine, increasing JP2 protein expression in a rat PH model [65]. These results indicate that increased JP2 or JP2NT expression is safe and feasible for the treatment of related diseases.

In addition to the change in JP2 expression level related to disease, according to the gene analysis results in patients with heart diseases, it has been confirmed that there are gene mutations in JP2 in some heart diseases, such as *S101R*, *Y141H*, *T161L*, *S165F*, and *E169K* mutations in the MORN region; *A399S*, *403S*, and *A405S* mutations in the *α*-helix structure; and *R436C* and *G505S* mutations in the divergent domain [74–79].

## 5. JP2 Function in Vascular Smooth Muscle

JP2 mRNA has been detected in vascular smooth muscles [12]. Pritchard et al. found that JP2 was the main junctophilin subtype of cerebral artery smooth muscle cells (CASMCs). Acute JP2 knockdown reduced JMCs between the SR and the PM; however, it did not affect the frequency, amplitude, and dynamics of spontaneous Ca^2+^ sparks produced by CICD but almost eliminated the Ca^2+^ spark activation, big conductance, and Ca^2+^-activated K^+^ (BK) channel currents. BK channels activate potassium efflux, cause hyperpolarization, inhibit voltage-gated calcium channels, and cause vasodilation. JP2 contributed to maintaining the functional coupling between RyR2s and BK channels, maintaining normal potassium outflow and normal cerebral artery contraction and relaxation [53]. Thus, JP2 knockdown inhibited cerebral artery relaxation and enhanced cerebral artery contraction. Saeki et al. confirmed that the joining region of JP2 (271–290 residues) interacted with caveolin-1 (Cav 1) and located BK channels near RyRs in mouse mesenteric artery smooth muscle cells (mMASMCs) to mediate the crosstalk between RyRs and BK channels. It can also transform local Ca^2+^ sparks into membrane hyperpolarization, which stabilizes the normal tension of mMASMCs [52]. Furthermore, JP2 can also interact with TRPC3 [87], which is responsible for ROCC in PASMCs, as shown in Figure 2 [7, 8]. Therefore, JP2 may interact with TRPC3 to maintain calcium homeostasis, thereby affecting the function of smooth muscle cells (SMCs) function.

## 6. JP2 and PH

PH is characterized by an increase in mPAP, pulmonary vascular contraction, pulmonary vascular remodeling, and right ventricular hypertrophy. According to JP2 function on the vascular smooth muscle, JP2 interacts with Cav 1 to form peripheral coupling and mediates RyRs that activate BK channels to induce PM hyperpolarization in SMCs to stabilize their resting tone [52, 53]. JP2 knockdown inhibits cerebral artery relaxation and enhances cerebral artery contraction [53]. Furthermore, we also found that JP2 can be cleaved by calpain under stress. The increase in [Ca^2+^]_i_ in PASMCs reportedly promotes calpain expression and activity, which contribute to the development of PH. In 2012, Ahmad et al. used the narrow-spectrum calpain inhibitor MDL-28170 to treat PH and reported that it mitigated the severity of right ventricular cardiomyocyte contractile dysfunction and attenuated right heart failure in acute PH. However, it has no effect on some recognized troponin substrates (including troponin, desmin, and hemoglobin), and the mechanism of its hemodynamic benefit is unclear [82]. JP2 is also a substrate of calpain. Therefore, we speculate that MDL-28170 may protect PASMC structure and function by inhibiting JP2 cleavage. In 2017, Prins et al. found that colchicine depolymerized microtubules increased JP2 and improved right ventricular function in monocrotaline-induced PH. Moreover, it enhanced the right ventricular pulmonary artery coupling and reduced pulmonary vascular disease severity [65]. These results suggest that JP2 is a reasonable target for the treatment of PH.

## 7. Drugs Targeting JP2 on PH

JP2 affects calcium signaling in the vascular smooth muscle and is correlated with PH. Therefore, it is feasible to consider JP2 as a new therapeutic target for PH. Based on the function and mechanisms described above, we believe that there are four strategies targeting JP2 for PH treatment. First, MDL-28170, an inhibitor of calpain, may attenuate right heart function in PH by maintaining JP2 expression and T-tubule stability [82]. It has also been confirmed that L-arginine intake can reduce calpain activity through S-nitrosylation, thus weakening Ca^2+^ regulatory protein proteolysis and deficiency [83]. The selective MMP inhibitor ARP-100 can improve cardiac contractility, attenuate junctophilin-2 proteolysis, and prevent JMC damage in the I/R-injured heart [60]. Second, puerarin is an isoflavone extracted from *Pueraria lobata*. Wang et al. reported that puerarin increased JP2 transcription and promoted T-tubule formation in cardiomyocytes [84]. In addition, *Smilax glabra* flavonoids and Icariin which are traditional Chinese medicines can promote JP2 expression in cardiomyocytes [85, 86]. Therefore, these three traditional Chinese medicines may be used to treat PH by increasing JP2 expression. Third, JP2 overexpression has an obvious curative effect on failure hearts [57, 86]. JP2NT is a transcription factor that can inhibit the transcription of key regulators of hypertrophy, fibrosis, and inflammation, which are also causes of vascular remodeling in PH, and JP2NT overexpression did not affect baseline cardiac morphology or function [26]. Thus, JP2NT overexpression may be a better choice for the treatment of PH. Fourth, as mentioned above, the subcellular localization of JP2 is mediated by microtubules. Therefore, both microtubule depolymerizers and stabilizers such as nocodazole, colchicine, and paclitaxel can improve JMC damage [33].

More and better JP2 specific inhibitors can be developed with the deepening of research. Of course, because the side effects of these strategies are unclear, their clinical application requires additional research support.

## 8. Conclusion

JP2 is a critical component of JMCs for E-C coupling in myocardial and smooth muscle cells; it is an essential component of calcium signal transduction. Damage can lead to cell dysfunction and clinical disease. This paper reviewed the basic functions of JP2 and its related diseases and mechanisms. Based on the characteristics of PH and the research progress of JP2 in SMCs in recent years, the latest use of JP2-targeting drugs in the treatment of PH has achieved good results, and this study speculated that it is feasible to improve PH by targeting JP2, thus providing a theoretical basis for future research.

However, the exact role of JP2 in PAMSC remodeling in patients with PH requires further study. For example, it remains unclear whether JP2 expression is decreased in PAMSCs of PH patients by transcription or degraded by protease; whether there are any changes in the JP2 protein modification pattern in the pathological process PH, such as phosphorylation, palmitoylation, ubiquitination, and glycosylation; whether the JP2 mutation causes PH; what the effect of JP2 targeting is on PH; how JP2 can be targeted in PASMCs; and how we can develop peptides such as JP2NT to replace transgenic therapy. Therefore, the specific role and mechanism of JP2 in PH requires more comprehensive and systematic study in the future. This is also of great significance for the development of new drugs for it.

## Data Availability

The data used to support the findings of this study are available from the corresponding author upon request.
